# Reverse total shoulder arthroplasty for proximal humerus fractures as an outpatient procedure

**DOI:** 10.1016/j.jsea.2026.100075

**Published:** 2026-08-11

**Authors:** Samuel Lorentz, Nicole Rivera, Eoghan Hurley, Alyssa Henriquez, Olivia Blaber, Bradley Fox, Tally E. Lassiter, Oke Anakwenze, Christopher S. Klifto, Michael A. Moverman

**Affiliations:** Department of Orthopaedic Surgery, Duke University, Durham, NC, USA

**Keywords:** Total shoulder arthroplasty, Outpatient, Proximal humerus fracture, Reverse total shoulder arthroplasty for fracture, Reverse total shoulder arthroplasty, Shoulder trauma

## Abstract

**Background:**

The purpose of this study is to compare short-term outcomes, including complication rates, readmissions, and reoperations, between inpatient and outpatient reverse total shoulder arthroplasty (rTSA) performed for proximal humerus fractures.

**Methods:**

All rTSA performed for proximal humerus fractures from January 2020 to January 2025 were retrospectively analyzed. Patients were evaluated based on whether they had the surgery performed as an inpatient (at least 1 overnight hospital stay) or as an outpatient (discharged on the same day as surgery). The decision for inpatient versus outpatient surgery was based on the surgeon's discretion. Complications within the first 30 and 90 days, including readmission and reoperation, were compared between the 2 groups, and a *P* value of <0.05 was considered to be statistically significant.

**Results:**

A total of 131 rTSAs for proximal humerus fractures were performed during the study period. Fifty-four patients underwent outpatient surgery and 77 patients underwent inpatient surgery. Average age in the outpatient group was 72.0 ± 6.8 years compared to inpatient 75.1 ± 9.7 years (*P* = .043). There were no significant differences in remaining patient demographics. Of the surgeries completed in the inpatient setting, the average length of stay was 4.75 ± 5.9 (range 1-44 days). There were no significant differences in the rates of emergency department (ED) visits that may be reasonably related to surgery within 90 days (18.5% vs. 18.2%; *P* = 1.000). Common reasons for ED visits included pain, wound complications, gastrointestinal diagnoses, and urinary tract infections. Patients who underwent outpatient rTSA showed no statistically significant difference in 90-day readmission rates compared to inpatient rTSA (3.7% vs. 13.0%; *P* = .121). Common reasons for readmission included cardiopulmonary complications. No patient in either cohort required further surgical intervention in the first 90 days following surgery.

**Conclusion:**

rTSA for proximal humerus fractures can be safely performed in the outpatient setting for appropriately selected patients, with no significant differences in 30-day or 90-day ED visits, readmission, or reoperations compared to inpatient management. Careful patient selection, incorporating medical and social factors, may allow for expanded use of outpatient pathways while maintaining patient safety.

Reverse total shoulder arthroplasty (rTSA) has emerged as the preferred surgical intervention for displaced proximal humerus fractures, particularly in elderly patients.^7^ Studies have shown superior functional outcomes compared to alternative surgical options, with improvements in validated outcome measures including the Constant and American Shoulder and Elbow Surgeons scores.^7^^,^^10^ Concurrently, there has been a significant increase in rTSA utilization for proximal humerus fractures. By 2019, rTSA surpassed the rate of internal fixation as the most common operative intervention for older adults, despite no overall increase in the percentage of proximal humerus fractures treated surgically.^18^ McLean et al^14^ further demonstrated that rTSA use increased from 4.1% to 24.5% of all proximal humerus fractures treated between 2008 and 2017, demonstrating the rapid adoption of this procedure.

The shift in surgical management is occurring in a population characterized by advanced age, greater comorbidity burden, and higher resource utilization. Patients with proximal humerus fractures require substantial perioperative support, and increasing comorbidity burden has been associated with up to 65% longer hospital stays for those undergoing operative treatment.^12^ Conversely, non-operative management in this population has been linked to higher readmission rates and hospital costs.^9^^,^^12^ As a result, rTSA for fractures has historically been performed in the inpatient setting.^11^ Furthermore, rTSA for acute proximal humerus fractures represents a more complex episode of care than elective rTSA, with longer operative times, increased blood loss, and higher rates of discharge to extended care facilities.^11^

O'Donnell et al^17^ reported on the accelerated growth in outpatient total arthroplasty, increasing from 3% of all total shoulder arthroplasties in 2019 to 38% in 2022. Multiple studies have demonstrated that elective outpatient shoulder arthroplasty can be performed safely without increased complications or readmissions while significantly reducing health-care costs.^1^^,^^3^^,^^5^^,^^8^ Systematic reviews have shown that outpatient total shoulder arthroplasty is associated with low rates of failed same-day discharge (0.9%), readmissions (3.9%), and complications (7.6%), with cost reductions ranging from $3,600 to over $50,000 per patient.^3^^,^^4^ Appropriate patient selection is essential for safe outpatient shoulder arthroplasty, but the majority of literature focuses on elective shoulder arthroplasty rather than acute fracture cases, which come with different risk profiles and medical complexity.

Despite the typical inpatient management of these patients, limited literature exists evaluating outpatient rTSA specifically for acute proximal humerus fractures. The purpose of this study is to compare short-term outcomes, including complication rates, readmissions, and reoperations, between inpatient and outpatient rTSA performed for proximal humerus fractures. The hypothesis was that outpatient rTSA for proximal humerus fracture will not be associated with increased short-term complications compared to inpatient procedures.

## Materials and methods

### Study design

All rTSA for proximal humerus fractures performed between January 2020 and January 2025 at a single tertiary referral institution were retrospectively analyzed. Institutional review board approval was granted for this study. Inclusion criteria consisted of all primary reverse total shoulder arthroplasties performed for acute proximal humerus fractures during the study period, with 90-day follow-up. Patients were evaluated based on whether they had the surgery performed as an inpatient (at least 1 overnight hospital stay) or as an outpatient (discharged on the same day as surgery). The decision for inpatient versus outpatient surgery was based on the surgeon's discretion. All patients in this study underwent interscalene nerve block.

### Data collection

Patient demographics and perioperative characteristics were collected, including age, sex, ethnicity, smoking status, length of stay, and dates of admission and discharge. Additionally, American Society of Anesthesiologists (ASA) physical status classification, Charlson Comorbidity Index, estimated intraoperative blood loss, and anesthesia type were collected for each patient. Complications within the first 30 and 90 days following surgery, including emergency department (ED) visits, readmission, and reoperation, were compared between the 2 groups. All ED visits and readmissions were individually evaluated through chart review and categorized as related to the index procedure.

### Statistical analysis

Standard statistical tests were used to compare the cohorts: Student t test for continuous variables and Pearson χ2 test for categorical variables. *P* < .05 was considered statistically significant. All data were analyzed using Microsoft Excel software (version 16.71; Microsoft, Redmond, WA, USA).

## Results

### Demographics and cohort characteristics

Between 2020 and 2025, 131 patients met the inclusion criteria. The mean age was 73.8 ± 8.7 years, and 25 patients (19%) were male, while 106 (81%) were female. There were 54 patients who underwent outpatient rTSA and 77 patients who were treated inpatient. Patients in the inpatient group were older than in the outpatient group (75.1 ± 9.7 vs. 72.0 ± 6.8 years, *P* = .043). There were no other significant differences in patient demographics between groups (Table I). Inpatient patients had a significantly higher ASA classification than outpatient patients (median 3 [interquartile range 2–3] vs. median 2 [interquartile range 2–3]; *P* = .013). There were no significant differences in Charlson Comorbidity Index (1.7 ± 1.6 vs. 1.8 ± 2.2; *P* = .488), estimated intraoperative blood loss (177 ± 72 vs. 190 ± 123 mL; *P* = .432), or anesthesia type between groups (*P* = .967).

**Table I:** Patient demographics and cohort characteristics.

Patient characteristics	Outpatient (n = 54)	Inpatient (n = 77)	*P* value
Age, yr, mean ± SD	72.0 ± 6.8	75.1 ± 9.7	.043∗
Gender M/F	11/43	14/63	.823
Smoker, %			
Current	7.4	6.5	1.000
Former	37.0	39.0	.857
Never	51.9	53.2	1.000
Not assessed	3.7	1.3	.569
Length of Stay	n/a	4.75 ± 5.9	n/a
ASA classification, median [IQR]	2 [2–3]	3 [2–3]	.013∗
Charlson Comorbidity Index, mean ± SD	1.8 ± 2.2	1.7 ± 1.6	.488
Estimated blood loss, mL, mean ± SD	190 ± 123	177 ± 72	.432
Anesthesia type, %	Gen + Reg 66.7%/Gen 27.8%	Gen + Reg 67.5%/Gen 26.0%	.967

*Yr*, year; *SD*, standard deviation; *ASA*, American Society of Anesthesiologists; *IQR*, interquartile range; *Gen + Reg*, general + regional; *Gen*, general only; *n/a*, not applicable.

∗ Indicates statistical significance.

Outpatients were discharged on the same calendar day as surgery. Inpatients had a mean length of stay of 4.75 ± 5.9 days (range 1-44 days) (Table I).

### Post-operative outcomes

Within 30 days of surgery, there were no significant differences in ED visits between the inpatient and outpatient cohorts (11.7% vs. 14.8%, *P* = .608). Similarly, 30-day readmission rates were comparable between inpatient and outpatient groups (6.5% vs. 3.7%, *P* = .699). At 90 days post-operatively, overall ED visits remained similar (inpatient 18.2% vs. outpatient 18.5%, *P* = 1.000). No statistically significant difference was detected in 90-day readmission rates between outpatient and inpatient cohorts (3.7% vs. 13.0%, *P* = .121) (Table II). The most common reasons for ED presentation included pain, wound concerns, and cardiopulmonary symptoms. Readmissions were frequently related to cardiopulmonary complications. No patients in either group required reoperation within 90 days of surgery.

**Table II:** 30-d and 90-day complications.

ED/Readmission	Outpatient (n = 54)	Inpatient (n = 77)	*P* value
ED visits within 30 d of surgery	8 (14.8%)	9 (11.7%)	.608
Readmission within 30 d	2 (3.7%)	5 (6.5%)	.699
ED visits within 90 d of surgery	10 (18.5%)	14 (18.2%)	1.000
Readmissions within 90 d	2 (3.7%)	10 (13.0%)	.121

*ED*, emergency department; *d*, days.

## Discussion

The most important finding of this study is that rTSA for proximal humerus fracture can be safely performed in the outpatient setting in appropriately selected patients. There were no significant differences in 30-day or 90-day ED visits or readmissions between inpatient and outpatient cohorts. Within 90 days, no patients required reoperation.

The majority of the patients in this cohort underwent inpatient management. The inpatient group was significantly older than the outpatient group (75.1 vs. 72.0 years, *P* = .043). Multiple factors contribute to the decision-making of inpatient versus outpatient management of proximal humerus fractures. Age and frailty are well-recognized factors influencing admission decisions in proximal humerus fractures, as older patients often have greater comorbidity burden and diminished physiological reserve.^15^^,^^22^ Prior studies have shown that frailty is common in this population and is associated with increased post-operative complications.^22^ Williamson et al^22^ demonstrated that frail patients have a 3-times higher 30-day complication risk following proximal humerus surgery, with the revised risk analysis index showing superior predictability for complications compared to other frailty indices. In studies of outpatient shoulder arthroplasty for elective indications, age above 70 years has been identified as a significant predictor of unplanned overnight hospital stay (odds ratio 36.80) and unplanned ED visits (odds ratio 7.52).^19^ However, other studies have shown that outpatient shoulder arthroplasty can be safely performed in patients aged ≥65 years without increased complications, suggesting that chronological age alone should not be an absolute contraindication.^21^

Concerns regarding medical optimization, pain control, mobility, and social support frequently contribute to conservative admission practices.^13^ In addition to medical considerations, non-clinical factors such as insurance status, geographic region, hospital type, and surgeon preference also influence admission decisions.^15^ Social determinants of health, including living situation, social support, and economic status, have been shown to significantly impact outcomes after proximal humerus fracture treatment. Patients living with family or friends prior to injury have shorter hospital stays and lower rates of new discharge destinations compared to those living alone.^13^

Despite this historical preference toward inpatient management, especially for older patients, post-operative outcomes in this study were the same between groups. Rates of both 30-day and 90-day readmissions were numerically lower in the outpatient cohort, although these differences were not statistically significant. These findings suggest that selected patients may safely undergo outpatient management of rTSA for proximal humerus fractures, though no statistically significant difference in outcomes was detected and firm conclusions are limited by study design. Our results add to emerging evidence supporting the safety of shoulder arthroplasty in the outpatient setting, including both anatomic and rTSA procedures.^1^^,^^3^^,^^5^^,^^8^ Although frailty and comorbidity burden are discussed as relevant factors influencing outpatient selection in this population, these variables were not formally measured or captured in the current cohort, which represents an important limitation in interpreting the discussion of frailty-related evidence relative to our findings. Similar trends have been observed in studies evaluating inpatient and outpatient open reduction and internal fixation for proximal humerus fractures, which similarly demonstrate no significant differences in short-term complications between the 2 groups.^2^ In our study, inpatient versus outpatient management was determined by surgeon discretion, reflecting real-world clinical decision-making regarding admission. Despite this, no differences in outcomes were observed; however, there are no validated criteria for which proximal humerus fracture patients are appropriate for outpatient management.

The findings of this study suggest that with appropriate patient selection, outpatient rTSA for proximal humerus fractures can be performed safely. Key considerations for patient selection should include age, medical comorbidities, ASA classification, tobacco use, social support, and living situation.^6^^,^^13^^,^^19^ Pre-operative optimization strategies, including anesthetic protocols, multimodal pain management, and patient education, are essential components of successful outpatient programs.^20^ Additionally, ensuring adequate post-operative support, including access to physical therapy, caregiver availability, and proximity to medical care, may help prevent complications and unplanned readmissions.^13^^,^^16^ Future research should evaluate how frailty indices, comorbidity profiles, and social determinants of health can be incorporated into evidence-based criteria for safe outpatient selection.

### Limitations

This study has several limitations. Its retrospective design introduces the potential for selection bias, especially considering that inpatient versus outpatient designation was determined by surgeon preference rather than standardized criteria. The relatively small sample size and single-center nature limits statistical power and generalizability. In particular, the relatively small sample size and low frequency of rare events such as readmission and reoperation may have resulted in insufficient power to detect clinically meaningful differences between groups, raising the possibility of type II error. As a consequence of the study design, we are unable to evaluate functional outcomes, pain scores, or patient-reported outcome measures, which are important components of post-operative recovery. Additionally, unmeasured confounders such as frailty status, social support, and discharge disposition were not captured and may have influenced admissions decisions and post-operative outcomes. Despite these limitations, this study provides preliminary evidence supporting the safety of outpatient rTSA for proximal humerus fractures in appropriately selected patients and highlights the need for prospective studies incorporating standardized risk stratification and functional outcome assessment.

## Conclusion

rTSA for proximal humerus fractures can be safely performed in the outpatient setting for appropriately selected patients, with no significant differences in 30-day or 90-day ED visits, readmission, or reoperations compared to inpatient management. Careful patient selection incorporating medical and social factors may allow for expanded use of outpatient pathways while maintaining patient safety.

## Disclaimers:

Funding: No funding was disclosed by the authors.

Conflicts of interest: Tally E. Lassiter has a consulting relationship with Lima and Stryker and research support from Arthrex and Wright Medical Technology.

Oke Anakwenze is a consultant for Exactech and SurtureTech.

Christopher S. Klifto has a consulting relationship with Smith and Nephew, restor3d, Stryker, and Accumed.

Michael A. Moverman has a consulting relationship with DJO.

Any additional authors, their immediate families, and any research foundations with which they are affiliated have not received any financial payments or other benefits from any commercial entity related to the subject of this article.
